# Non-alcoholic Fatty Liver Disease Among Iraqi Patients With Psoriasis: A Case-Control Study

**DOI:** 10.7759/cureus.57487

**Published:** 2024-04-02

**Authors:** Samer A Dhaher, Noora Z Hilfi, Muntadher A Abdullah

**Affiliations:** 1 Dermatology, College of Medicine, University of Basrah, Basrah, IRQ; 2 Internal Medicine, Basrah Gastroenterology and Hepatology Hospital, Basrah, IRQ

**Keywords:** fibrosis, steatosis, fibroscan, metabolic syndrome, nafld, psoriasis

## Abstract

Background: Non-alcoholic fatty liver disease (NAFLD) is the most prevalent chronic liver disease worldwide. Several studies have shown that patients with psoriasis have a higher risk of developing NAFLD. Obesity, metabolic syndrome, and cytokine-mediated inflammation might be the link between psoriasis and NAFLD.

Aims: This study aims to investigate the prevalence of NAFLD among psoriatic Iraqi patients and examine the relationship with disease severity using the Psoriasis Area and Severity Index (PASI) score and the correlation with different clinical and laboratory parameters.

Subjects and methods: A case-control study on 130 psoriatic patients and 130 age-, sex-, and BMI-matched healthy controls was conducted at the Department of Dermatology in Basra Teaching Hospital from November 2022 to October 2023. All demographic and clinical data were collected using a pre-designed questionnaire, and NAFLD was diagnosed through a FibroScan examination performed on each participant. The severity of psoriasis was determined using the PASI score. Fasting glucose, liver enzymes, and lipid profile levels were investigated, and metabolic syndrome was identified.

Results: The prevalence of NAFLD was significantly higher in our psoriatic patients than in the control group (66.2% vs. 42.3%, OR=2.6, P<0.01). Psoriatic patients were found to have more severe NAFLD than the controls, as evidenced by their steatosis and fibrosis staging (P<0.01). In patients with psoriasis, NAFLD was associated with a higher prevalence of diabetes (17.4%) and metabolic syndrome (55.8%). Furthermore, psoriatic patients with NAFLD had significantly higher values of BMI, waist circumference, PASI score, as well as serum alanine transaminase (ALT), triglyceride, cholesterol, low-density lipoprotein (LDL), and fasting glucose levels. The study also found a significant positive correlation between the psoriasis severity based on PASI and the steatosis score. Metabolic syndrome, PASI, BMI, serum triglycerides, LDL, and age are the independent predictors of NAFLD.

Conclusions: NAFLD is highly prevalent among psoriatic patients affecting more than half of them and closely associated with metabolic syndrome and severity of psoriasis. Routine screening for NAFLD may be necessary in psoriatic patients particularly when considering the use of hepatotoxic drug therapy.

## Introduction

Psoriasis is a chronic inflammatory and proliferative dermatosis that affects a significant portion of the population. Genetic susceptibility, immune-mediated inflammation, and environmental factors all play a role in this disease. The prevalence of the disease among adults varies significantly by country, ranging from 0.91% in the United States to 8.5% in Norway [1]. The reported prevalence in Iraq is 2.3% [2]. Psoriasis affects both adult women and men equally, with onset typically occurring before the age of 40. The age of onset tends to follow two peaks, with one between 20 and 30 years and another between 50 and 60 years [3]. Patients with psoriasis are susceptible to a variety of comorbidities, including psoriatic arthritis in about one-third of patients [4], risk of cardiovascular disease (CVD) [5-7], and metabolic syndrome (MetS) in 14.3-50% of patients [8,9]. Obesity is a common comorbidity of psoriasis, and several studies have shown that patients with psoriasis are more frequently overweight or obese compared with patients without psoriasis [10]. Non-alcoholic fatty liver disease (NAFLD) is defined as the excessive accumulation of fat in the liver (hepatic steatosis) without any other identifiable causes, such as excessive alcohol consumption, viral hepatitis, certain medications like methotrexate, steroids, amiodarone, and tamoxifen, as well as other inherited or acquired disorders like Wilson's disease and lipodystrophy [11]. NAFLD is the most prevalent chronic liver disease worldwide, affecting 30% of the population. It is highly associated with metabolic risk factors such as obesity, diabetes mellitus, and dyslipidemia in most patients [12]. Prior researches have documented a substantial occurrence of NAFLD among patients with psoriasis, with prevalence rates ranging from 17% to 65% [13-17]. Accordingly, patients with psoriasis are at a higher risk of developing NAFLD, especially among patients with severe psoriasis [18]. In a large prospective population-based study including 2292 participants, the prevalence of NAFLD was 46% in 118 patients with psoriasis and 33% in individuals without psoriasis [16]. In line with these findings, a systematic review and meta-analysis of seven case-control studies discovered that patients with psoriasis had a higher risk of NAFLD than a control group with no psoriasis (OR=2.15) [19]. Given the complex genetic, immunological, and environmental factors involved, the relationship between NAFLD and psoriasis remains incompletely understood. Both NAFLD and psoriasis are multifactorial diseases that share common exposomes such as internal factors like oxidative stress due to an imbalance between the production and accumulation of reactive oxygen species in cells and tissues and the body's ability to detoxify these reactive substances and microbiome imbalance, which trigger the production of the IL-17 cytokine that fuels simultaneously the pathogenic pathways of psoriasis. External contributors of exposome that also promote psoriasis and NAFLD include general external factors (climate, biodiversity, urban environment, social, and economic elements) and specific external factors (infections, allergens, diet, tobacco, pollutants, and toxic substances) [20]. The aim of this study was to examine the potential association between NAFLD and psoriasis in our population, which has not been previously explored. To the best of our knowledge, no prior research has been conducted on this subject. Therefore, our objectives were to investigate the prevalence of NAFLD among a population of Iraqi patients with psoriasis and determine if there is any correlation between the degree of NAFLD and psoriasis severity, patient demographics, and metabolic parameters.

## Materials and methods

Study design

A case-control study was conducted at the Department of Dermatology in Basrah Teaching Hospital, Basrah Governorate, Iraq, from November 2022 to October 2023. In total, 260 participants were enrolled: 130 patients with psoriasis and 130 age-, sex-, and BMI-matched, apparently healthy, randomly selected controls.

Inclusion and exclusion criteria

Patients aged 18 years and above with plaque psoriasis were included. In contrast, people who had hepatitis B or C (shown by a positive blood test), had inflammatory or autoimmune diseases, were pregnant or breastfeeding, had a history of cancer or chemotherapy, were currently on systemic therapy, or had received methotrexate, acitretin, cyclosporine, biologics, systemic steroids, or drugs or habits that could alter patient BMI like beta-blockers, sulphonylureas, injectable progestins, and smoking for at least six weeks before enrollment were not eligible.

Ethical consideration and data collection

A formal consent was obtained from each participant, and ethical approval was obtained from the Ethical Committee of the College of Medicine, University of Basrah (approval number: 030408-023-2022). The demographic data and detailed medical history were recorded. All participants underwent evaluation for BMI by using the following equation: BMI=weight (kg)/height (m^2^). Waist circumference and blood pressure were measured. The Psoriasis Area and Severity Index (PASI) was used to assess the severity of psoriasis (the PASI score ranges from 0 to 72; a PASI score less than 7 was mild psoriasis, a PASI score of 7-12 was moderate, and a PASI score of more than 12 was severe) [21].

Laboratory investigation

Following an overnight fast, blood samples were taken from each patient in the morning to evaluate liver enzymes, cholesterol, triglycerides, low-density lipoprotein (LDL), high-density lipoprotein (HDL), and fasting blood sugar (FBS). The following values are interpreted as normal: alanine transaminase (ALT)=4-36 U/L, aspartate aminotransferase (AST)=8-33 U/L, cholesterol <200 mg/dL, triglyceride <150 mg/dL, LDL <100 mg/dL, HDL ≥50 mg/dL in females and ≥40 mg/dL in males, and FBS <100 mg/dL [21,22]. Based on the updated National Cholesterol Education Program (NCEP) Adult Treatment Panel III (ATPIII) definition, metabolic syndrome was defined as having at least three of the following signs as shown in Table 1 [23].

**Table 1:** Criteria for the clinical diagnosis of metabolic syndrome HDL: high-density lipoprotein; BP: blood pressure

Measure (any three of five is diagnostic of metabolic syndrome)	Categorical cut points
Elevated waist circumference	≥102 cm in men
	Or ≥88 cm in women
Elevated triglyceride	≥150 mg/dL
	Or on treatment for elevated triglyceride
Reduced HDL	<40 mg/dL in men
	Or <50 mg/dL in women
	Or on treatment for reduced HDL
Elevated BP	Systolic BP ≥130 mmHg
	Or diastolic BP ≥85 mmHg
	Or on antihypertensive drugs
Elevated fasting glucose	≥100 mg/dL
	Or on treatment for elevated glucose

Diagnosis of NAFLD

Transient elastography was used to measure liver steatosis and fibrosis with FibroScan® (FibroScan® Compact 530, Echosens, Paris, France). At Basrah Gastroenterology and Hepatology Hospital, a skilled hepatologist performed the test. Based on several prior research studies and references provided by the manufacturer, the following steatosis and fibrosis stages were identified as shown in Table 2 [24,25].

**Table 2:** Staging of steatosis and fibrosis according to FibroScan measurement

FibroScan findings	Staging	Value
Steatosis	S0	<238 dB/m
	S1	238-260 dB/m
	S2	261-290 dB/m
	S3	>290 dB/m
Fibrosis	F0	>5.8 kPa
	F1	5.8-6.8 kPa
	F2	6.9-7.8 kPa
	F3	7.9-11.8 kPa
	F4	>11.8 kPa

Statistical analysis

The adequate sample size was estimated using a simple formula for the case-control study (available at https://shorturl.at/anpES) with a level of confidence of 95%, a power of 80%, and a type I error of 5%. Considering that the prevalence of NAFLD is 30% in the population based on previously published data [12], a sample size of at least 119 participants is required for each group. Statistical analysis was performed using IBM SPSS Statistics for Windows, Version 26.0 (Released 2019; IBM Corp., Armonk, New York, United States). Categorical variables were presented as numbers and percentages, and continuous variables were presented as means with standard deviations (SD). The chi-squared test evaluated the association between categorical variables. An independent sample t-test was used to compare two means. The correlation between psoriasis severity based on PASI and steatosis score was analyzed using Pearson's correlation test. Logistic regression analysis was used to test the association between psoriasis and NAFLD, and the results were expressed as odds ratios (OR) and 95% confidence intervals (95% CI). For all analyses, a p-value <0.05 was considered statistically significant.

## Results

The demographic characteristics of patients and controls who participated in this study are summarized in Table 3. Out of 130 psoriatic patients, 85 (65.4%) were males and 45 (34.6%) were females with a mean age of 38.8±13.1 years, ranging from 18 to 74 years compared to 130 apparently healthy controls, 77 (59.2%) males and 53 (40.8%) females, with a mean age of 36.9±11.1 years ranging from 18 to 70 years. The differences between the two groups were statistically not significant, with a p-value >0.05. Regarding BMI and waist circumference, there were no significant differences between the two groups (p>0.05).

**Table 3:** Demographic characteristics of the study population P<0.05 is considered significant

Variables		Patients (n=130)	Control (n=130)	P-value
Age (years)	Range	18-74	18-70	0.216
	Mean±SD	38.8±13.1	36.9±11.1	
Sex, no. (%)	Male	85 (65.4%)	77 (59.2%)	0.306
	Female	45 (34.6%)	53 (40.8%)	
BMI (mean±SD)		28.3±4.7	27.8±4.04	0.413
Waist circumference (cm) (mean±SD)		93.2±11.4	90.7±13.9	0.122

The mean duration since psoriasis diagnosis was 8.6±7.2 years (range: six months to 30 years). Diabetes and hypertension were reported by 17 (13.1%) and 13 (10%) of patients, respectively. The PASI score showed that 62 patients (47.7%) had severe psoriasis, 25 (19.2%) had moderate, and 43 (33.1%) had mild disease. Furthermore, metabolic syndrome was found in 43.1% of the cases.

Table 4 presents the laboratory parameters of psoriatic patients. The study found that 30 (23.1%) patients had elevated FBS, while 29 (22.3%) and 27 (20.8%) patients had elevated ALT and AST levels, respectively. Additionally, 66 (50.8%) had high serum cholesterol, and 73 patients (56.2%) had high LDL levels. Almost half of the psoriatic patients had hypertriglyceridemia as well as low HDL levels.

**Table 4:** The laboratory parameters of psoriatic patients Data presented as mean±SD or number and percentage FBS: fasting blood sugar; ALT: alanine aminotransferase; AST: aspartate aminotransferase; LDL: low-density lipoprotein; HDL: high-density lipoprotein

Variables	Mean±SD level	Elevated level no. (%)
FBS (mg/dL)	101.3±24.3	30 (23.1%)
Serum ALT (U/L)	28.07±15.3	29 (22.3%)
Serum AST (U/L)	26.3±9.9	27 (20.8%)
S. triglyceride (mg/dL)	158.9±87.4	61 (46.9%)
S. cholesterol (mg/dL)	204.8±45.9	66 (50.8%)
S. LDL (mg/dL)	108.5±27.9	73 (56.2%)
S. HDL (mg/dL)	45.1±18.2	62 (47.7%)

The prevalence of NAFLD diagnosed by FibroScan was significantly higher in psoriatic patients compared to matched controls (66.2% versus 42.3%, P<0.01, OR=2.66, 95% CI: 1.6-4.4). Furthermore, psoriatic patients had higher mean values of steatosis and fibrosis scores in comparison to the control group (steatosis: 266.18±53.5 dB/m versus 233.33±20.85 dB/m, fibrosis: 4.93±1.67 kPa versus 3.87±0.78 kPa) with statistically significant differences (P<0.01) (Table 5).

**Table 5:** Prevalence of NAFLD and FibroScan measurements in psoriatic patients and control group Data presented as mean±SD or as number and percentage; P<0.05 was considered significant NAFLD: non-alcoholic fatty liver disease; OR: odds ratio; CI: confidence interval; dB/m: decibels per meter; kPa: kilopascal

Variable	Patients (n=130)	Control (n=130)	P-value	OR (95% CI)	
NAFLD, N (%)	86 (66.2%)	55 (42.3%)	<0.01	2.66 (1.6-4.4)	
No NAFLD, N (%)	44 (33.8%)	75 (57.7%)	<0.01		
Steatosis score (dB/m) mean±SD	266.18±53.50	233.33±20.85	<0.01		
Fibrosis score (kPa) mean±SD	4.93±1.67	3.87±0.78	<0.01		

According to their steatosis and fibrosis staging, psoriatic patients had more severe NAFLD than the controls (Table 6). Severe steatosis (S3) was found in 49 (37.7%) psoriatic patients with NAFLD, but not in any of the NAFLD controls. On the other hand, the majority of NAFLD cases in the control group (45 (34.6%)) were stage 1 steatosis (S1). According to liver fibrosis staging, most of the studied population showed either no fibrosis (93 (71.5%) of patients and 127 (97.7%) of the controls) or mild fibrosis (24 (18.5%) of patients and two (1.5%) of the controls). None of the participants in both groups had severe fibrosis (F4). Stage 3 fibrosis (F3) was found only in six (4.6%) psoriatic patients with NAFLD, but not in any of the controls. This difference was statistically significant (P<0.01).

**Table 6:** Steatosis and fibrosis staging in psoriatic patients versus controls Data presented as number and percentage; P<0.05 was considered significant

Test	Stage	Patients	Controls	P-value
Stages of steatosis, N (%)	S0	44 (33.8%)	75 (57.7%)	<0.001
	S1	17 (13.1%)	45 (34.6%)	
	S2	20 (15.4%)	10 (7.7%)	
	S3	49 (37.7%)	0 (0%)	
Stages of fibrosis, N (%)	F0	93 (71.5%)	127 (97.7%)	<0.001
	F1	24 (18.5%)	2 (1.5%)	
	F2	7 (5.4%)	1 (0.8%)	
	F3	6 (4.6%)	0 (0%)	
	F4	0 (0%)	0 (0%)	

When comparing psoriatic patients with NAFLD to psoriatic patients without NAFLD, we found that those with NAFLD were older and more obese, with a higher BMI and waist circumference than those without NAFLD, showing a significant difference (P<0.05). In addition, psoriatic patients with NAFLD had a higher prevalence of diabetes compared to the other group. However, there were no significant differences between the two groups in terms of disease duration, mean systolic and diastolic blood pressures, or the prevalence of hypertension. Furthermore, those with NAFLD had more severe psoriasis, as assessed by the PASI score, compared to those without NAFLD (16.6±10.7 versus 9.9±8.9, P<0.01). The study found that those with NAFLD had considerably higher levels of FBS, ALT, total serum cholesterol, triglycerides, and LDL than those in the same group who did not have NAFLD. The NAFLD group also reported higher serum AST levels and lower HDL levels, although they did not achieve statistical significance. NAFLD patients had a higher prevalence of metabolic syndrome than non-NAFLD patients (P<0.01) (Table 7).

**Table 7:** Comparison of demographic, clinical, and laboratory characteristics between psoriatic patients with NAFLD and without NAFLD Data presented as mean±SD or as number and percentage; P<0.05 was considered significant NAFLD: non-alcoholic fatty liver disease

Variables	Psoriasis with NAFLD (n=86)	Psoriasis without NAFLD (n=44)	P-value
Age (years), mean±SD	40.9±12.1	34.8±14.2	0.012
Duration of psoriasis (years), mean±SD	9.27±7.5	7.5±6.6	0.19
BMI (kg/m^2^), mean±SD	29.9±4.6	25.2±3.1	0.001
Waist circumference (cm), mean±SD	96.1±9.9	87.5±12.3	0.001
Systolic (mmHg), mean±SD	123.5±8.9	120.6±8.5	0.077
Diastolic (mmHg), mean±SD	80.07±6.8	77.3±9.8	0.065
Diabetes, N (%)	15 (17.4%)	2 (4.5%)	0.039
Hypertension, N (%)	10 (11.6%)	3 (6.8%)	0.387
PASI, mean±SD	16.6±10.7	9.9±8.9	0.001
FBS (mg/dL), mean±SD	106.08±28.3	92.02±7.1	0.003
ALT (U/L), mean±SD	30.6±16.6	23.03±11.02	0.007
AST (U/L), mean±SD	27.43±10.1	24.2±9.3	0.084
Cholesterol (mg/dL), mean±SD	214.3±44.7	186.2±41.9	0.002
Triglyceride (mg/dL), mean±SD	181.3±90.5	115.09±61.1	0.001
LDL (mg/dL), mean±SD	116.06±28.8	93.8±19.1	0.001
HDL (mg/dL), mean±SD	43.4±18.3	48.4±17.5	0.135
Metabolic syndrome, N (%)	48 (55.8%)	8 (18.2%)	0.001

Logistic regression analysis was performed to identify the parameters that independently impact NAFLD in psoriatic patients; the study found that BMI, PASI score, metabolic syndrome, FBS, triglycerides, and LDL were independent predictors of NAFLD (Table 8).

**Table 8:** The association of NAFLD with different demographic and laboratory parameters in psoriatic patients P<0.05 was considered significant OR: odds ratio; CI: confidence interval; BMI: body mass index; WC: waist circumference; PASI: Psoriasis Area and Severity Index; FBS: fasting blood sugar; ALT: alanine aminotransferase; AST: aspartate aminotransferase; LDL: low-density lipoprotein; HDL: high-density lipoprotein

Variables	P-value	OR	95% CI
Age	0.04	1.04	1.001-1.08
Sex	0.137	2.7	0.72-10.02
BMI	0.006	2.99	1.37-6.51
WC	0.183	1.04	0.98-1.1
Diabetes	0.345	2.35	0.39-13.8
Hypertension	0.696	0.71	0.137-3.77
Duration of psoriasis	0.272	1.04	0.96-1.12
PASI	0.004	2.2	1.29-3.78
FBS	0.02	1.06	1.009-1.13
Cholesterol	0.414	1.005	0.99-1.016
LDL	0.01	1.02	1.007-1.05
HDL	0.98	1.00	0.97-1.02
Triglyceride	0.02	1.01	1.001-1.019
ALT	0.848	1.004	0.96-1.04
AST	0.622	0.98	0.92-1.04
Metabolic syndrome	0.001	5.32	2.1-13.4

**Figure 1:**
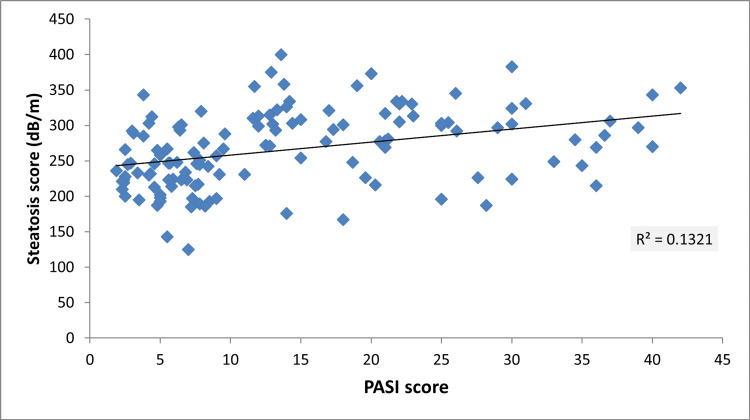
Scatter plot diagram shows the correlation between steatosis score and PASI score in psoriatic patients PASI: Psoriasis Area and Severity Index

Furthermore, the study found a significant positive correlation between NAFLD based on steatosis score and the severity of psoriasis based on PASI score with r=0.363 and p-value=0.001 (Figure 1).

## Discussion

Psoriasis is a common inflammatory skin disease that has been associated with various comorbidities. Studies have shown that individuals with psoriasis are more likely to have NAFLD. This increased risk of NAFLD is attributed to a higher incidence of metabolic syndrome and insulin resistance, which promote the development of hepatic steatosis [26]. This risk is particularly significant because people with psoriasis frequently require hepatotoxic drugs, such as methotrexate, which may worsen underlying liver disease. Identifying the risk factors for NAFLD in this population is crucial for implementing screening and treatment protocols for high-risk psoriatic patients. In the current study, 130 psoriatic patients and 130 matched controls were compared, and we found that the prevalence of NAFLD was substantially greater in our psoriatic patients than in the control group (66.2% vs. 42.3%). This estimate is in line with the findings of an Iranian case-control study [17], which showed that the prevalence of NAFLD in psoriasis was 65.6%, whereas that of matched controls was 35%. Another cross-sectional study carried out in Indonesia found a greater frequency of NAFLD (77.8%), which was connected with the severity of psoriasis [26]. In the outpatient adult US population, psoriasis was linked to NAFLD (32% vs. 26.6%), according to another study by Ruan et al. [27]. According to a study conducted in Spain by Belinchón-Romero et al., 42.3% of psoriatic patients had NAFLD [14]. However, a pilot study carried out in India discovered that there was no statistically significant difference in the prevalence of NAFLD between matched healthy controls and psoriatic patients [28]. The observed discrepancies in NAFLD prevalence across several studies could likely be ascribed to variations in the initial features and clinical contexts, such as age, disease severity, and the existence of metabolic syndrome. The fact that psoriatic patients had more severe NAFLD than the control group was another significant finding in this study. Their significant fibrosis and steatosis scores demonstrated this, which is in line with a previous study by Abedini and colleagues [17]. Given that both TNF-α and IL-6 are known to contribute to the onset and progression of NAFLD, the cause of this is most likely the elevated levels of both cytokines in psoriasis. Moreover, research has demonstrated that the single factor linked to cirrhosis and hepatic fibrosis is TNF-α [28,29]. Low-grade chronic inflammation, peripheral insulin resistance, and elevated oxidative stress are common features of both psoriasis and NAFLD. In the early phases of NAFLD, insulin resistance and hyperinsulinemia play a crucial role in stimulating the release of circulating free fatty acids, which is then followed by the abnormal buildup of triglycerides in hepatocytes and hepatic steatosis. This may be followed by a phase in which simple steatosis transforms into steatohepatitis due to an enhanced inflammatory response [30]. There may be a bi-directional relationship between psoriasis and NAFLD through pro-inflammatory pathways, proposed as the hepato-dermal axis. Upon reaching the liver, pro-inflammatory cytokines such as TNF-α and IL-17 derived from psoriatic epidermis may influence hepatic inflammation and insulin resistance. In psoriasis, pro-inflammatory mediators originating from hepatic inflammation may contribute to the development or exacerbation of cutaneous inflammation [31].

In the current study, we examined the relationship between various clinical and laboratory parameters as predictors for the increased risk of having NAFLD in psoriatic patients, and we discovered that metabolic syndrome was the strongest predictor of having NAFLD (OR=5.32, 95% CI: 2.1-13.4, P<0.01), followed by BMI (OR=2.99, 95% CI: 1.37-6.51, P<0.01) and PASI score (OR=2.2, 95% CI: 1.29-3), while the other variables did not show a statistically significant association. It is important to note that this study found a significant positive correlation between NAFLD and the severity of psoriasis based on the PASI score. Although Gandha et al. reported no significant correlation between NAFLD and PASI, they did find a significant positive correlation between NAFLD and body surface area as a measure of disease severity [32]. This finding is interesting since it shows that the risk of NAFLD is proportional to the severity of psoriasis. As a result, it is crucial to screen patients with severe psoriasis for NAFLD before initiating systemic therapy.

The study has certain limitations. First of all, because the study was conducted at a single center, our data cannot be fully generalized to the total population. Second, because the study was cross-sectional in nature and had a short study period, it was challenging to determine a causative and reciprocal association between psoriasis and NAFLD. However, this problem can be addressed by conducting larger prospective studies with longer follow-up periods to corroborate our findings.

## Conclusions

Psoriatic patients have a higher prevalence of NAFLD than do healthy controls in our population. Age, triglyceride, LDL, BMI, FBS, metabolic syndrome, and PASI score were independent predictors of NAFLD. Patients with psoriasis, especially those with metabolic syndrome, may require routine screening for NAFLD using FibroScan, and this is particularly crucial when it comes to the prescription of potentially hepatotoxic drugs for the treatment of psoriasis. We need prospective studies with larger sample sizes to find out how psoriatic treatments, especially systemic medications like methotrexate and biologics, affect the development and course of NAFLD. We also need to find out if psoriasis and NAFLD are mutually linked by looking at the occurrence of psoriasis in NAFLD patients.
